# Human iPSC derived disease model of MERTK-associated retinitis pigmentosa

**DOI:** 10.1038/srep12910

**Published:** 2015-08-11

**Authors:** Dunja Lukovic, Ana Artero Castro, Ana Belen Garcia Delgado, María de los Angeles Martín Bernal, Noelia Luna Pelaez, Andrea Díez Lloret, Rocío Perez Espejo, Kunka Kamenarova, Laura Fernández Sánchez, Nicolás Cuenca, Marta Cortón, Almudena Avila Fernandez, Anni Sorkio, Heli Skottman, Carmen Ayuso, Slaven Erceg, Shomi S. Bhattacharya

**Affiliations:** 1 CABIMER (Centro Andaluz de Biología Molecular y Medicina Regenerativa), Avda. Americo Vespucio s/n, Parque Científico y Tecnológico Cartuja, 41092, Sevilla, Spain; 2 Stem Cell therapies in Neurodegenerative diseases Lab, and National Stem Cell Bank-Valencia Node, Biomolecular and Bioinformatics Resources Platform PRB2,ISCIII, Research Center “Principe Felipe”, c/ Eduardo Primo Yúfera 3, 46012, Valencia, Spain; 3 Department of Physiology, Genetics and Microbiology, University of Alicante, Campus de San Vicente del Raspeig, 0369 Alicante, Spain; 4 Department of Genetics and Genomics, IIS-Fundación Jiménez Díaz, 28040 Madrid, Spain; 5 Center for Biomedical Network Research on Rare Diseases (CIBERER), ISCIII, Madrid, Spain; 6 BioMediTech, University of Tampere, Biokatu 12, 33520 Tampere, Finland

## Abstract

Retinitis pigmentosa (RP) represents a genetically heterogeneous group of retinal dystrophies affecting mainly the rod photoreceptors and in some instances also the retinal pigment epithelium (RPE) cells of the retina. Clinical symptoms and disease progression leading to moderate to severe loss of vision are well established and despite significant progress in the identification of causative genes, the disease pathology remains unclear. Lack of this understanding has so far hindered development of effective therapies. Here we report successful generation of human induced pluripotent stem cells (iPSC) from skin fibroblasts of a patient harboring a novel Ser331Cysfs*5 mutation in the MERTK gene. The patient was diagnosed with an early onset and severe form of autosomal recessive RP (arRP). Upon differentiation of these iPSC towards RPE, patient-specific RPE cells exhibited defective phagocytosis, a characteristic phenotype of MERTK deficiency observed in human patients and animal models. Thus we have created a faithful cellular model of arRP incorporating the human genetic background which will allow us to investigate in detail the disease mechanism, explore screening of a variety of therapeutic compounds/reagents and design either combined cell and gene- based therapies or independent approaches.

Retinitis pigmentosa (RP; OMIM 268000) with a prevalence of 1 in 3,500 individuals is the most common form of hereditary retinal disorder affecting the working age group. RP is characterized by progressive dysfunction and death of mainly the rod photoreceptor cells (PR) of the retina however in some cases retinal pigment epithelium (RPE) cells are also involved, often resulting in permanent blindness. So far 54 genes have been implicated in this disease coding for proteins involved in a myriad of functions such as phototransduction signaling cascade, retinoid cycle, cell-cell adhesion or the cytoskeleton^1^. The disease is inherited in all there Mendelian forms, the autosomal recessive (arRP) being the most common with over 50% of cases. Largely due to the high genetic heterogeneity and unavailability of disease tissue, pathology of the disease remains elusive.

Patient-derived induced pluripotent stem cells (iPSCs) provide an unprecedented opportunity to recapitulate disease pathogenicity without the need for genetic manipulation and creation of gene targeted animal models. Human iPSCs, similar to embryonic stem cells (ESC), can be expanded indefinitely *in vitro* and differentiated into any type of mature cell in the human body, without the ethical and immunogenicity issues associated with ESC^2^. These cells are also valuable for developing therapeutic strategies, drug toxicity screens and development of disease models, in addition to providing a source for cell transplantation therapy. RPE cells and photoreceptors (PR) have been successfully generated from iPSCs (iPSC-RPE and iPSC-PR respectively) by various groups in stepwise differentiation protocols mimicking retinal development by introducing Wnt signaling inhibitors (DKK1), Nodal antagonist Lefty A, Notch pathway inhibitor (DAPT-gamma secretase inhibitor), or IGF-1^3^,^4^,^5^. In contrast, only RPE cells have been generated spontaneously in overgrown iPSC/ESC cultures without the addition of exogenous factors, since derivatives of neuroectoderm appear by default in non-induced cultures^6^,^7^. Generated RPE cells in these studies display a fully mature phenotype and physiological activity *in vitro* such as phagocytosis, secretion of vascular endothelial growth factor (VEGF) and pigment epithelium-derived factor (PEDF) and epithelial barrier formation. Cellular models of hereditary retinal dystrophies have been successfully created *in vitro* in Best disease, and RP where patients’ fibroblasts were reprogrammed to iPSC and then converted to RPE^8^,^9^ or photoreceptor cells^10^, expressing the disease phenotype. iPSC- derived RPE (iPSC-RPE) cells have also been shown to have a protective effect when injected sub-retinaly into the Royal College of Surgeons (RCS) rats^11^ and RPE65-defective mice^12^. Moreover, iPSCs have met clinical-grade requirements^13^ as a source of RPE grafts and have recently been injected in patients affected by the exudative form (wet-type) of age-related macular degeneration (AMD)^14^. It has been argued that in this form of AMD the dysfunction and loss of RPE cells is the main cause of visual impairment in the elderly.

Mer tyrosine kinase receptor (MERTK) belongs to the Tyro3/Axl/Mer (TAM) receptor tyrosine kinase family of proteins distinguished by a conserved intracellular kinase domain and extracellular adhesion molecule-like domain. TAM receptors regulate a variety of processes such as cell proliferation/survival, adhesion, migration, inflammatory response, in a cell- microenvironment- and ligand- specific manner^15^. In previous studies *MERTK* was found to be disrupted in RCS rats^16^,^17^, a classic model for retinal degeneration inherited as an autosomal recessive trait, and found to cause early- onset retinitis pigmentosa in patients^18^. RPE cells fail to phagocytize the shed outer segment (OS) material of PR, a circadian activity performed by RPE cells which serves to renew the damaged lipid and protein components of light exposed PR, while new membranous discs are formed (disc biogenesis) and inserted in the basal part of the OS. As a result, RCS rats exhibit OS associated debris accumulation in the subretinal space, abnormal OS length, eventually leading to the onset of PR degeneration by the P20 stage. Usually complete degeneration occurs by P60. Similar phenotype is observed in *mer*^*kd*^ mice^19^ indicating that the RPE phagocytic defect is the underlying molecular mechanism of disease in humans carrying *MERTK* mutations. Indeed, the reduced retinal thickness and debris detected in the sub-retinal space in patients harboring the *MERTK* –splice-site -mutation resembles the observed phenotype in the RCS rat^20^. The distinctive clinical presentation of RP is the only disease manifestation of patients harboring *MERTK* mutations without any systemic disease or defects of phagocytosis by macrophages, indicating a specialized function of this protein in the RPE cells. In contrast to the detailed clinical understanding of the disease, the mechanism by which MERTK acts during the phagocytosis remains partially unveiled. Outer segments are known to bind to the integrin receptor αvβ5^21^ followed by focal adhesion kinase (FAK) activation in the apical membrane of RPE^22^ while the MERTK activation occurs via Gas6/Protein S, TUB, TULP1 ligand binding^23^,^24^. The latter is thought to activate autophosphorylation at tyrosine Y-749, Y-753 and Y-754 in the tyrosine kinase domain, which in turn activates the molecular cascade targeting actin or non-muscle myosin II to coordinate the cytoskeletal rearrangements necessary for phagocytic ingestion^25^.

In this study we describe the generation of an *in vitro* model of RP caused by the mutation of *MERTK* gene using human iPSC technology. We report the generation of a cellular model of MERTK-associated RP, which recapitulates the disease phenotype described in animals and in patients and provides a tool to advance our understanding of the signaling pathways activated by MERTK. Importantly this can be studied in detail in the context of the human genetic background, a crucial feature of the cellular model. Equally important is that it provides us with a unique opportunity for the testing of novel therapeutic agents in the presence of the human background, which has the potential to significantly reduce the cost of preclinical studies in animal models. The derivation of iPSCs from a RP patient’s fibroblasts and the generation of a RPE cell line expressing the molecular defect makes an important contribution in our ability to undertake translational research.

## Results

### RP caused by *MERTK* gene mutation

Skin punch biopsies were taken from a phenotypically well-characterized RP patient and an unaffected individual after the signing of informed consent. Forty-five- year-old patient born of a consanguineous marriage (patient II:2; family RP-0503, Supplementary Fig. S1A) was diagnosed with early-onset autosomal recessive RP (arRP). Genome-wide linkage analysis using the Illumina HumanCyto-12 SNP array followed by homozygosity mapping was performed in this Spanish family with 3 affected and 2 unaffected siblings that revealed a large region of 32 Mb with a maximum logarithm of the odds (LOD) score of 3.6 on chromosome 2 encompassing *MERTK*. Sequence analysis allowed identification of a novel homozygous frameshift mutation c.992_993delCA (p.Ser331Cysfs*5) in *MERTK*, segregating with the disease in the family (Supplementary Fig. S1A).

All affected members of this family were diagnosed suffering from RP during the first decade of life. Patient II:2 complained of night blindness, loss of peripheral visual field and visual acuity (VA) at the age of 7. Ophthalmological examination at 32 years of age showed severe constriction of visual fields in both eyes and loss of VA to 20/70 in right eye and finger counting in the left eye. The fundus showed typical changes of RP with pale disc, narrowed retinal vessels and bone-spicule pigmentations in the peripheral retina. For this study, a healthy individual unrelated to this family and no family history of blindness was taken as a healthy control. The healthy individual had a VA of 20/20 at the time of skin biopsy.

**Figure 1:**
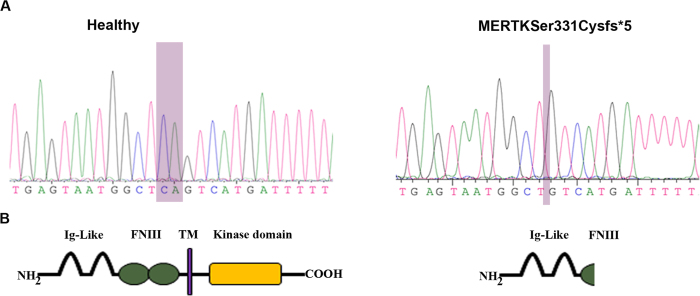
(**A**). *MERTK* DNA sequence chromatogram around c.992_993 in healthy and affected patient carrying a deletion of CA (c.992_993delCA). DNA sequencing, performed on healthy and patient’s dermal fibroblasts and iPSCs’ gDNA, confirmed the mutation in early-onset RP patient. (**B**). The wild type sequence codes for 999 amino acid protein with Immunoglobulin Ig-like C2-type (Ig-like) (residues 100–194 and 203–281), fibronectin type III-like (FN3) (residues 284–368 and 384–470) transmembrane (TM) (residues 502–524) and kinase domain (residues 587–854). The mutation results in a predictive truncated protein of 334 amino acids with expected molecular weight of 36.6 kDa.

Primary fibroblast cell lines were established from the patient II:2 and healthy individual in parallel and subjected to direct genomic sequencing to confirm the genotype (Fig. 1A). The deletion c.992_993delCA was confirmed in the patient’s DNA. The *MERTK* gene is composed of 19 coding exons with two immunoglobulin-like C2-type (Ig-like) domains, two fibronectin type III (FN3)-like domains, a transmembrane (TM) domain and a tyrosine kinase (TK) domain (UniProt Q12866). The deletion of CA in exon 7 (NM_006343.2), at position 992_993 causes the frameshift resulting in a premature stop codon at position 335 (Supplementary Fig. S1B). The predictive stop codon results in a truncated protein with 2 Ig-like domains and part of the first FN3-like domain with no kinase activity (Fig. 1B).

### Generation of iPSC from healthy individual and RP patient

The primary fibroblasts derived from the skin biopsies were expanded for three to-five passages before being reprogrammed by a Sendai viral construct containing four genes: Oct3/4, Sox2, Klf4 and cMyc (Supplementary Fig. S2A). This construct described as highly efficient in transduction was selected for being non-integrative and replication deficient. Thirty days after transduction, iPSC colonies were selected by their morphology (refractive edges, high nuclear/cytoplasmic ratio) and *in situ* staining of pluripotency markers (TRA-1-60 or TRA-1-81). Three iPSC lines per individual were sub-cultured and analyzed at cellular and genetic level to confirm successful reprogramming. Pluripotency was assessed by immunocytochemistry for pluripotency markers such as octamer-binding transcription factor 4 (OCT4), NANOG, SRY (sex determining region Y)-box 2 (SOX2), and *in situ* staining to TRA-1-81. The alkaline phosphatase is known to be more active in iPSCs and the colorimetric assay depicting its activity confirmed that the selected iPSC colonies are indeed pluripotent (Supplementary Fig. S3A). To test the ability of generated iPSC lines to generate derivates of three germ layers *in vivo,* the iPSCs were transplanted subcutaneously into the immunodeficient (SCID) mice. Eight weeks after injection, tumor was formed and extracted. Histological sections showed that the tumor contained derivatives of all three germ layers including gut-like tissues (endoderm), neural cells and retina (ectoderm) and striated muscle, bone, cartilage and adipose tissue (mesoderm) (Supplementary Fig. S3B). The healthy individual’s iPSCs were indistinguishable from patient’s iPSCs with respect to pluripotency characterization. All selected lines were transgene-free at passages 7–10 and karyotypically normal over 30 passages (Supplementary Fig. S2B and C, respectively). DNA fingerprinting was performed with iPSC lines and proved their genetic identity to parental fibroblasts (Supplementary Fig. S2D).

### Differentiation of iPSCs into RPE cells

**Figure 2:**
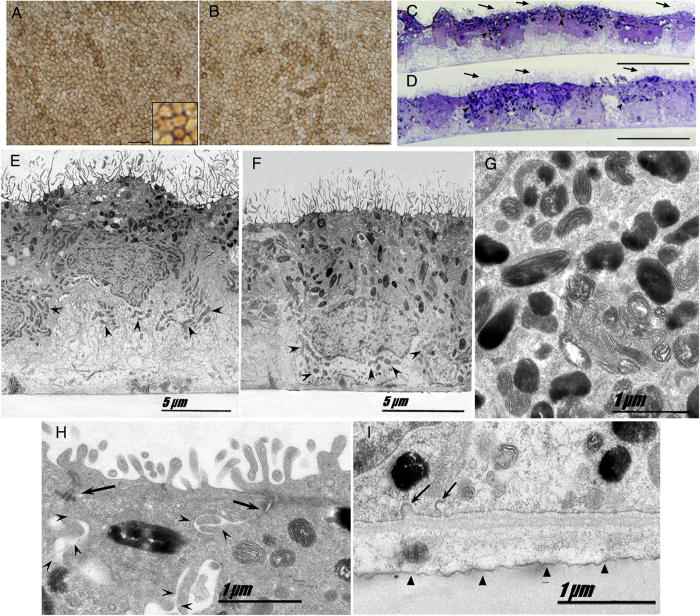
Differentiation of healthy and MERTK p.Ser331Cysfs*5 iPSCs into RPE cells. Bright field micrograph of healthy (**A**) and MERTK p.Ser331Cysfs*5 (**B**) iPSC-RPE showing typical RPE morphology, polygonal shape and pigmentation. Semithin sections of iPSC-RPE cells stained with toluidine blue. Both healthy (**C**) and MERTK p.Ser331Cysfs*5 (**D**) iPSC-RPE cells form a monolayer of cuboid cells highly polarized with abundant apical microvilli (arrows) and melanosomes (arrowheads). Electron micrograph of cultured iPSC-RPE, healthy (**E**) and MERTK p.Ser331Cysfs*5 (**F,G,H,I**). (**G**) High magnification of melanin granules showing different stages of development. (**H**) Intercellular junctional complexes which include the tight junctions, adherens junctions (arrows) and membrane interdigitations (arrowheads). (**I**) The basement membrane of the cells tightly bound to the transwells support film (arrowheads) and small basal infoldings (arrows). A,B scale bar 50µm; C,D scale bar 10µm.

We induced patient’s and the healthy individual’s iPSCs to differentiate toward RPE cells in parallel, by removing the pluripotency factor bFGF and culturing the cells in suspension until dark patches appeared in the floating aggregates as previously described^6^ (Supplementary Fig. S4). The pigmented areas of the aggregates were mechanically excised, trypsinized and plated as a monolayer cell culture. After 3–4 weeks in culture the iPSC-derived RPE (iPSC-RPE) cells appeared to exhibit polygonal, cobblestone-like morphology, which is characteristic of mature RPE cells. The areas with correct morphology were lifted and reseeded on permeable culture inserts to yield a uniform cellular monolayer. To reach full functional maturity, the cells were cultured for additional 30–80 days until high pigmentation. Figure 2A,B depicts the mature iPSC-RPE cells from both individuals showing indistinguishable polygonal morphology and pigmentation. Toluidine blue staining (Fig. 2C,D) and transmission electron microscopy of the iPSC-RPE cells (Fig. 2E,F) show that the apical surface of the cell monolayer is at the top, where abundant microvilli can be observed. Melanosomes are represented with black round and oval shapes (Fig. 2G) and have apical distribution. Mitochondria that are ellipsoidal in shape and seen below the nuclei are largely displaced toward the basal-lateral part of the cells (Fig. 2E,F arrowheads), the natural position for these organelles *in vivo.* Intercellular junctional complexes are visible in appropriately aligned sections of iPSC-RPE of both individuals. The integrity and function of the RPE monolayer depend on its basolateral cellular junctions, which include the tight junctions, adherens junctions and membrane interdigitations (Fig. 2H). The basement membrane of the cells is tightly bound to the transwells support film and small basal infoldings were observed (Fig. 2I).

**Figure 3:**
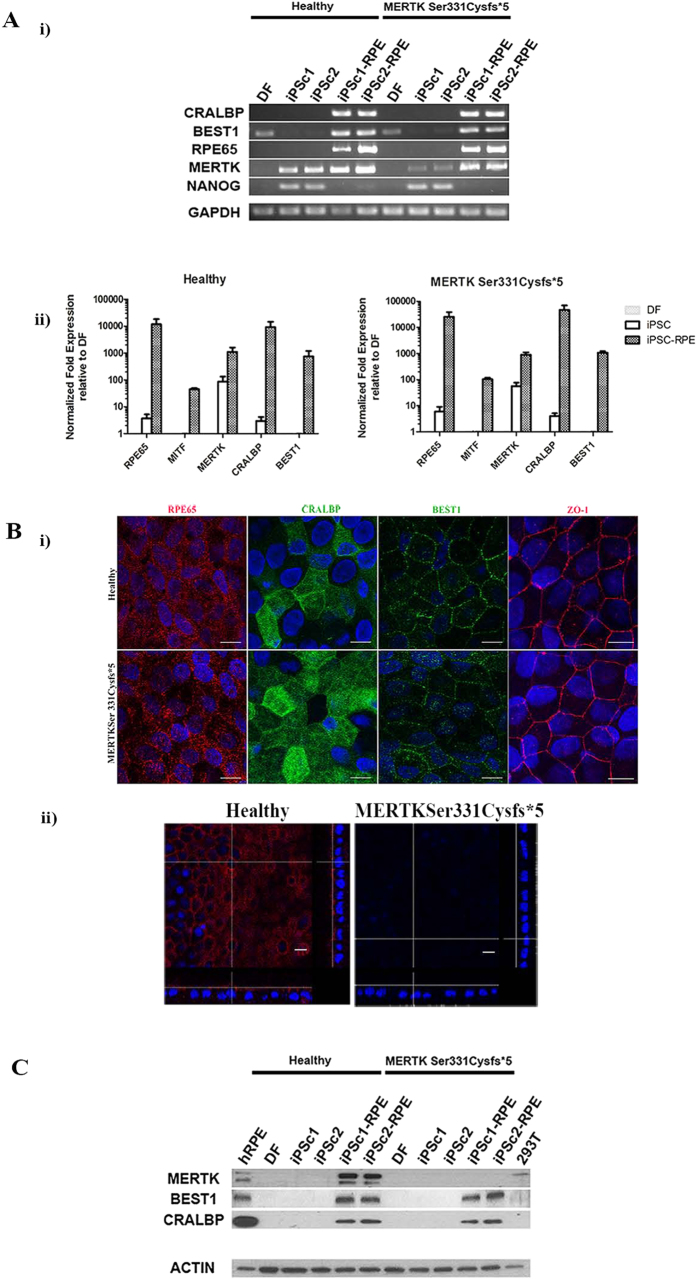
RPE characteristic marker gene and protein expression in iPSC-RPE from healthy individual and RP patient. (**A. i**) *BEST1*, *CRALBP*, *RPE65*, *MERTK* gene expression in fibroblasts, iPSCs and iPSC-RPE. Two iPSC lines (iPSCc1 and iPSCc2) and their respective iPSC-RPE from each individual are analyzed. The samples were loaded on agarose gels prepared and run under the same experimental conditions. (**ii**) Quantitative reverse transcription polymerase chain reaction to analyze expression of genes characteristic for RPE. Fold expression in undifferentiated iPSCs and iPSC-RPE from healthy and MERTK p.Ser331Cysfs*5 patient normalized to their originating dermal fibroblasts (DF). Each bar represents the average ±SEM of at least three independent biological replicates (* p ≤ 0.05, **p ≤ 0.05, ***p ≤ 0.05). (**B. i**) Expression of RPE characteristic proteins by iPSC-RPE. Immunocytochemistry against RPE65, CRALBP, BEST1, ZO-1 in healthy and MERTK p.Ser331Cysfs*5 iPSC-RPE. Images were taken with Leica confocal microscope TCS SP5 using HCX PL APO lambda blue 63X/ 1.4 OIL objective. Scale bar 10µm. (**ii**) MERTK expression in iPSC-RPE. Immunocytochemistry against MERTK. Apical section and vertical section simulation showing apical MERTK distribution in the healthy individual. MERTK p.Ser331Cysfs*5 iPSC-RPE stain negative for MERTK at all sections. Images were taken with Leica confocal microscope TCS SP5 using HCX PL APO lambda blue 63X/ 1.4 OIL objective, scale bar 10 μm. (**C**). Western blot analyses of RPE-specific marker protein expression in fibroblasts (DF), two iPSC lines (iPSCc1 and iPSCc2) and their respective iPSC-RPE from each individual are analyzed. The expression of CRALBP and BEST1 is detected only in iPSC- RPE and hRPE cells. β actin was used as loading control. Cropped blots are from gels run under the same experimental conditions and loaded with the same samples.

To validate the identity of generated RPE cells, RT-PCR of selected RPE specific genes was performed to compare the expression levels throughout the differentiation process (Fig. 3A,i)). This included markers such as cellular retinaldehyde-binding protein (CRALBP), Bestrophin 1 (BEST1), retinal pigment epithelium-specific protein 65kDa (RPE65), MERTK, and pluripotency marker NANOG. The expression profile was compared in healthy and MERTK p.Ser331Cysfs*5 patient’s fibroblasts, two iPSCs lines and their respective iPSC-RPE. Figure 3A,i) shows the expected pattern of expression for RPE transcripts of *BEST1*, *CRALBP* and *RPE65* with comparable strong expression levels in iPSC-RPE from both individuals while, as expected, being absent from iPSCs and fibroblasts. *BEST1* was an exception, which shows a weak expression in fibroblasts. NANOG, a pluripotency marker, is expressed in iPSCs but silenced after the cells differentiate into RPE. The data described in Fig. 3A (i) were corroborated by quantitative analyses (Fig. 3A (ii)). The expression of RPE specific genes *CRALBP*, *RPE65, BEST1* and microphthalmia-associated transcription factor (MITF) are raised over a 1000 fold in iPSC-RPE cells from both individuals with respect to the fibroblasts, while *CRALBP* and *RPE65* are just detectable by qRT-PCR in iPSCs.

On checking expression of *MERTK* by RT-PCR using primers spanning exons 14–17 and also by qRT-PCR assay using primers spanning exon junction 18–19 (Fig. 3A, Supplementary Fig. S5) indicate that *MERTK* mRNA is detectable in both healthy and MERTK p.Ser331Cysfs*5 iPSCs and rises over 1000 times with respect to the original fibroblasts in iPSC-RPE of both individuals. *MERTK* expression in iPSC has not been previously described and is in correlation with its expression in human ESC^7^. The level of expression of *MERTK* in healthy individual was higher than in MERTK p.Ser331Cysfs*5 patient. The difference in expression pattern of *MERTK* in iPSC-RPE from healthy individual and RP patient is expected since it is known that eukaryotes possess a nonsense-mediated mRNA decay pathway, which degrades mRNAs containing nonsense mutations before they are translated into nonfunctional polypeptides^26^. The possibility remains that alternative transcript that does not include the mutation site is amplified by the primers and probe used in the assay. In the case that RNA transcribed from the c.992_993delCA allele escapes nonsense mediated decay, a truncated protein of 334 amino acids (expected size of 36,6 kDa) lacking the kinase domain would be produced. Overall, we can confirm that the generated iPSC-RPE cell lines originated from two different individuals exhibit the mature RPE gene expression pattern and are indistinguishable at this point.

Immunocytochemical staining revealed a strong expression of the visual cycle markers CRALBP, RPE65, as well as BEST1 and zonula-occludens-1 (ZO-1), a tight-junction marker, indistinguishable in both individuals (Fig. 3B,i)). Apico-lateral and apical localization of ZO-1 and sodium/potassium-dependent ATPase (Na^+^/K^+^ ATPase), respectively, is a hallmark of RPE cells and was found in both healthy and MERTK p.Ser331Cysfs*5 iPSC-RPE cells (Supplementary Fig. S6).

### MERTK expression in iPSC-RPE

Immunocytochemical detection of MERTK in iPSC-RPE monolayer from healthy individual showed its expression apically as previously described^27^ (Fig. 3B,ii)). In contrast, iPSC-RPE cells derived from the RP patient carrying the mutation p.Ser331Cysfs*5 are completely deficient in MERTK (Fig. 3B,ii)). The antibody used detected the N-terminal fragment of the protein implying that any truncated protein retaining the N-terminal domain is absent in the iPSC-RPE from the RP patient. This data corroborates the patient-specific origin of generated iPSC-RPE monolayers and additionally confirms the correct polarization of generated RPE cells.

Expression of MERTK and mature RPE specific markers was confirmed by western blot throughout the differentiation process of two iPSC lines per genotype (Fig. 3C). The expression pattern of RPE-specific markers is matched with human RPE (hRPE) extract from cadaveric donor by loading the same total protein amount. Irrespective of the individual, whether healthy or RP patient, RPE-specific markers BEST1 and CRALBP are absent from fibroblasts and iPSCs but are detected in iPSC-RPE monolayers at similar levels. MERTK is detected as a double band, which migrates with molecular weight of 180 and 130 kDa in healthy individual’s monolayer and native RPE while being absent in iPSC-RPE from RP patient harboring the MERTK mutation. Human embryonic kidney cells (HEK 293T) are known to express MERTK^28^ as a single 180 kDa band while being negative for RPE markers. The MERTK expression is detected also in iPSC from control individual upon higher protein load (Supplementary Fig. S7). A 180 kDa band corresponding to MERTK is also described in human ESC and ESC-RPE^7^, supporting the view that MERTK is expressed in pluripotent stem cells and corroborating the identity of the 180 kDa band. The observed molecular weight of MERTK by western blotting does not correspond to the predictive molecular weight of 110 kDa of the 999 amino acid long MERTK. The protein was detected at significantly larger sizes ranging from 165–205 kDa in different cell types due to posttranslational modification such as glycosyation (14 glycosylation sites are detected in UniProt database), ubiquitination or phosphorylation^15^,^28^. The two bands corresponding to 180 and 130 kDa peptides (Fig. 3C), demonstrate that these two isoforms are specific to RPE as confirmed in hRPE extract as opposed to a single 180 kDa band observed in HEK 293T cells. The western blot confirms the absence of MERTK and any truncated protein preserving the N-terminal domain (Supplementary Fig. S7) in RP-affected patient corroborating the identity of the patient-derived iPSC-RPE and indicating that the defective, kinase lacking, MERTK is probably degraded in patient’s RPE.

### Patient’s iPSC recapitulate the disease phenotype

**Figure 4:**
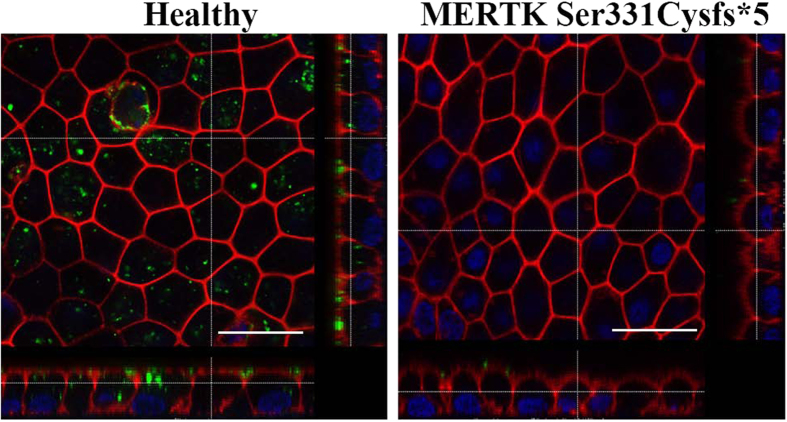
*In vitro* phagocytosis assay of photoreceptor outer segments (OS) by iPS-RPE from healthy individual and MERTK p.Ser331Cysfs*5 patient. Basolateral sections across the iPSC-RPE cells together with vertical section simulation. Healthy iPS-RPE internalize FITC-labeled OS (green) while patient’s one do not. F-actin is stained by phalloidin (red) to visualize cell morphology. Images were taken with Leica confocal microscope TCS SP8 using HCX PL APO lambda blue 63X/ 1.4 OIL objective, scale bar 25 μm.

MERTK deficient phenotype described in animal models is defective phagocytosis, a daily process of OS uptake, which renews the light–damaged membranes of PR. The process of OS phagocytosis can be mimicked in cultured RPE cells by feeding the isolated OS derived from bovine or pig retina^6^,^7^. We exposed the cultured iPSC-RPE monolayers from both individuals to fluorescently labeled bovine and pig OS and monitored their ingestion by confocal imaging. Figure 4 shows the internalization of OS by the iPSC-RPE from the healthy individual while this was completely lacking in the case of the RPE generated from the RP patient. The labeled phalloidin depicts the abundant F-actin in the microvilli and the OS are detected in the region of microvilli and inside the cells derived from healthy individual. Vertical sectioning demonstrating the ingested OS phagosomes inside the cells is shown on Supplementary Fig. S8. The patient’s iPSC-RPE monolayer showed only a few OS in the area of microvilli. Both iPS cell lines derived from the RP patient exhibited the same defect in phagocytosis of OS upon differentiation to RPE.

We have thus generated patient-specific RPE cells that lack the expression of MERTK and consequently recapitulate the MERTK deficient phenotype i.e. absence of phagocytosis. These data show that patient-specific, mature and functional RPE can be generated from control and RP patients via iPSCs. The monolayers derived from the two individuals as reported here are morphologically identical and indistinguishable by RPE-specific marker expression while preserving patient’s identity as observed by distinctive MERTK expression and its associated function.

## Discussion

Retinal dystrophies comprise a group of eye diseases caused by mutations in a spectrum of genes with a specific function in photoreceptors or RPE cells or in many cases mutations in ubiquitously expressed genes that exclusively give rise to a retinal phenotype. While retinal tissue is inaccessible for the studies of molecular mechanism, unrelated tissues such as blood is not truly representative of molecular events in the retina. Animal models remain an invaluable tool to understand disease mechanism, however in some cases they are not fully representative of the disease pathophysiology, owing to differences in neuronal development, physiology and the genetic background. In some cases, such as the Usher-1 mouse model, the animal lacks the disease phenotype^29^, or the *CHM* gene deficient mice (model for choroideremia) which are lethal^30^, iPSC-based models represent a viable alternative to study disease biology. Patient-specific iPSC offer a renewable cell source that can be coaxed toward a desired cell type, which offers a unique opportunity to gain valuable insight into the disease mechanism in addition to drug screening, toxicity testing and explore new therapies in the context of the human genetic background.

MERTK mutations account for approximately 1% of arRP and were reported in several families with retinal dystrophy^20^,^31^,^32^,^33^,^34^,^35^; with common clinical features such as early onset of symptoms, usually in the first decade of life, loss of peripheral vision as typically associated with rod-cone type of dystrophy and accumulation of OS by-product debris in the sub-retinal space detected by optical coherence tomography. These clinical parameters can be used as distinctive features of MERTK-associated RP facilitating early identification of patients. Early detection of patients should provide a better choice of therapeutic options. Altogether 20 mutations have been described in the *MERTK* gene causing RP, the majority being missense/nonsense mutations (11), splicing-site mutations (3), small deletions (3), insertions (1) and gross deletions (2) as reported in the HGMD public database (last accessed January 8, 2015). We show here the successful generation of iPSC from a RP patient bearing a novel p.Ser331Cysfs*5 mutation in MERTK which has not been described before. Adult dermal fibroblasts were reprogrammed with non-integrative virus delivering pluripotency genes and the generated iPSCs maintained genomic integrity throughout the culture period of over 30 passages. It should be noted that genomic alterations including numerical and structural alterations are not uncommon in iPSC cultures. In our experience the use of Sendai virus as a vehicle for gene delivery resulted in the absence of genomic alterations in contrast to integrative viral strategies. As demonstrated here, iPSCs established from the control and the affected patient were identical in relation to reprogramming efficiency and exhibited similar kinetics and efficiency of differentiation. Both iPSC-RPE were morphologically indistinguishable, the polygonal morphology and pigmentation was displayed by control and patient RPE. The RPE specific marker expression such as CRALBP, RPE65, BEST1 and ZO-1 was similar in both iPSC-RPE monolayers. A distinctive functional feature of the native RPE is the cellular polarity based on the polarized localization of RPE markers. We show polarized distribution of ZO-1, a tight junction marker, and Na^+^/K^+^ ATPase pump protein expressed on the apical membrane. This is a distinctive RPE feature since Na^+^/K^+^ ATPase is reported to be expressed basolaterally in other transporting epithelia^36^. MERTK distribution is also shown to be apical as previously described in RPE cells^27^. Another RPE functional characteristic is the presence of tight junctions essential for the formation of blood-retina barrier. We observe by electron microscopy structures corresponding to tight junctions and apico-lateral localization of ZO-1 by fluorescent microscopy. In conclusion it can be stated that MERTK deficiency does not interfere with these functional parameters.

In order to confirm that the difference between healthy and diseased cells is due to disease mutation and not due to inter-lineage variability described for iPSCs^37^,^38^ we studied in parallel two iPSC lines per individual. The disease-specific defect is then expected to be present in all disease iPSC lines while absent from all healthy iPSC lines which is confirmed by our findings. Analysis of the *MERTK* expression raises the possibility that the patient’s mRNA partially escapes nonsense–mediated decay and perhaps the transcript is translated into an abnormal 334 amino acid protein lacking the kinase domain. However, we do not detect the truncated protein in patient’s iPSC-RPE extracts by western blot nor by immunocytochemistry in the iPSC-RPE monolayer, suggesting that it may be degraded after being translated.

Our results prove that patient-specific iPSC reproduce the disease phenotype reliably and this should allow faithful modeling of degenerative disease of the RPE in the future with more subtle phenotypes and completely unknown disease mechanisms. In addition, the generated iPSC-RPE represents clinically reliable setting to test therapies in order to reverse or delay the disease onset in patients harboring *MERTK* mutations.

## Materials and Methods

### Genetic analysis

A consanguineous Spanish family (RP-0503) consisting of three affected siblings (Supplementary Fig. S1A) was recruited by the Fundacion Jimenez Diaz Hospital (Madrid, Spain). DNA was extracted from peripheral blood leukocytes collected in EDTA tubes using an automated DNA extractor (BioRobot EZ1; Qiagen; Hilden, Germany). Informed consent was obtained from all individuals involved, all procedures were reviewed and approved by the Ethics Committee of the Fundacion Jimenez Diaz Hospital and adhered to the tenets of the Declaration of Helsinki.

Whole-genome single nucleotide polymorphism (SNP) microarray analysis was performed using Illumina HumanCyto-12 SNP array, containing 298,199 SNPs markers (Illumina, Inc, San Diego, CA). Arrays were processed according to the manufacturer’s protocols. Linkage analysis was performed, using the Gene Hunter program (version 2.1r5) in the easyLINKAGE plus software package (version 5.08), to identify regions that may contain the causative mutation. As the affected siblings were born of a consanguineous marriage, only the homozygous regions were taken into account.

Bidirectional automatic sequencing was performed to screen the *MERTK* gene for mutations. Exons and exon–intron boundaries of *MERTK* were analyzed using oligonucleotide primer pairs designed using Primer3 software (http://frodo.wi.mit.edu/). Sequences and annealing temperatures are available from the authors on request. The PCR conditions and primer sequences are available on request. The PCR products were enzymatically purified with ExoSAP-it (USB, Affymetrix) and both strands sequenced using Big Dye Terminator Cycle Sequencing Kit version 3.1 (Applied Biosystems, Carlsbad, CA). The PCR products were purified in a 96-well multiscreen filter plate (Montage SEQ96 Sequencing Reaction Cleanup Kit; Millipore, Bedford, MA) and resolved on an automated sequencer (ABI 3130xl Genetic Analyzer, Applied Biosystems).

Direct sequencing in fibroblasts and iPSCs: Genomic DNA from fibroblasts and iPSCs was isolated using the QIAamp DNA Blood mini kit (Quiagen). Primers used for amplification and directed sequencing of *MERTK* upstream and downstream of c.992_993 were as follows: 5′CGAAGAGGTTCTAAGAGAGG3′ and 5′CCATTTTCATCAGTCGCCTC3′ (annealing temperature 55 °C).

### Derivation of skin fibroblasts

Skin biopsy of a healthy individual and an affected patient (with a confirmed molecular diagnosis of early-onset RP) was taken under sterile conditions following informed consent. The skin biopsy sample was placed in ~25 ml of sterile PBS at room temperature (20–25 °C) for shipment. Inside a tissue culture hood, the PBS was replaced with PBS containing 10× penicillin/streptomycin (500 U/ml penicillin and 500 μg/ml streptomycin) and 10× Fungizone (25 μg/ml amphotericin B) and left at room temperature for 15 min, with occasional mixing by inverting the tube. This step was repeated twice and finally the biopsy sample was washed with PBS for 15 min. The sample was cut into small pieces and cultured in 60-mm plastic dishes (8–10 pieces per dish) in DMEM (10% FBS, 2 mM GlutaMAX, 50 U/ml penicillin and 50 mg/ml streptomycin) at 37 °C under 5% CO_2_. After 3–4 weeks, fibroblasts outgrowing from the biopsy pieces cover most of the dish and cells were passaged and plated in a T-75 flask. After reaching 80% confluency fibroblasts were further expanded and cryostored in liquid N_2_.

### Generation and maintenance of iPSC lines

The iPSC were derived from patient’s fibroblasts using Sendai virus (Cytotune iPS reprogramming Kit, Life Technologies) according to manufacturer’s instructions. Single colony subcloning was performed for 5 passages and the presence of Sendai virus transgenes was detected by RT-PCR (Supplementary Fig. S2) with primers described in Supplementary Table S1, during passages 7–10. Cells set aside during reprogramming on day 7 were used as positive control. PCR was carried out using 500 ng of cDNA in the presence of MyTaq Red DNA Polymerase (Bioline) using manufacturer’s instructions. PCR products were analyzed using 2% agarose gel electrophoresis. iPSCs were grown on irradiated (45Gy) human foreskin fibroblasts (ATCC CRL 2429) in iPSCs medium containing KO DMEM, KSR 20%, Glutamax 2 mM, non essential aminoacids 0.1 mM, β-mercaptoethanol 0.23 mM, basic FGF 10 ng/mL, and peniciline/streptomicine. Cells were mechanically passaged every 6–8 days.

### Differentiation toward RPE cells

To obtain RPE cells the iPSCs colonies were mechanically removed and cultured in low attachment plates as floating aggregates in the iPSC medium with 15% KSR and deprived of bFGF. When dark patches formed on the aggregates they were mechanically excised, treated with trypsin and plated on Matrigel (BD, #354277) coated plastic culture dishes. Depending on the confluency of plating, 3–5 weeks later, monolayers of cells with RPE characteristics (polygonal shape, pigmentation) appeared. After excising the aggregated portions, the RPE-like monolayer was dissociated with trypsin–EDTA (0.05%), passed through a 70 μm strainer and plated at 200,000 cells/cm^2^ on Matrigel coated transwell inserts (Corning 3470, MilliCell). The RPE cells were cultured for 30–60 days until they reached high pigmentation. Two iPS cell lines per each genotype were induced to differentiate toward RPE and no differences were observed with respect to kinetics of differentiation, degree of pigmentation or characteristic RPE morphology between the iPS cell lines from one individual nor between healthy and affected genotype.

### Teratoma assay

For teratoma assay the colonies from a fully confluent 6-well plate were cut mechanically and resuspended in 200 μL of ihPSCs media and matrigel. The suspension was immediately injected into 5 week-old SCID nude mice subcutaneously on the dorsal part. After about 8 weeks, teratomas of 1 cm diameter were formed and these were excised. Teratomas were fixed in 4% PFA for 3 days, washed 3 times with PBS, sectioned in half and observed macroscopically for the presence of cysts. Then the samples were dehydrated using 70–80–95% ethanol and xylol and embedded in paraffin. Continuous 6 μm sections and hematoxilin/eosin staining were performed at the Histology Unit, CABIMER. Pictures were taken with Leica DM6000B, connected with Leica DFC 350 FX camera.

### Phagocytosis assay

Photoreceptor OS were obtained from InVision BioResources (WA, USA) or prepared in-house according to Molday *et al.*, 1987^39^. *OS labeling:* Two mg/ml stock solution of FITC isomer 1 (F7250, Sigma) in 0,1M sodium bicarbonate at pH 9.0–9.5, was prepared, filter-sterilized, and stored in aliquots at −20 °C. For labeling with FITC, OS were suspended in solution containing 10% sucrose, 20 mM sodium phosphate, and 5 mM taurine, and incubated with FITC for 1,5 h at room temperature, rotating in the dark. FITC-labeled OS were washed 5 times in buffer containing 10% sucrose, 20 mM sodium phosphate and 5 mM taurine, suspended in DMEM, aliquoted and stored at −80 °C until use.

iPSC-RPE cells were incubated with OS for 2 h/37 °C, after which the OS were removed by triple washing with PBS. Cells were fixed by 4% PFA for 15 min/RT, washed and permeabilized by 0.1% Triton-X. Phallodin staining was performed for 30 min, the samples were then mounted with vectashield mounting media with DAPI. The samples were visualized by Leica confocal microscope TCS SP5 using HCX PL APO lambda blue 63X/ 1.4 OIL objective.

### Transmission Electron Microscopy

RPE cultured cells were fixed in 4% paraformaldehyde, 2% glutaraldehyde in 0.1 M sodium phosphate buffer (pH 7,2–7,4) for 2 h, washed with the same buffer, and then postfixed in 1% OsO4 in PB. After gradual dehydration in ethanol series, the pieces were embedded in EPON 812. Semithin sections stained with 1% toluidine blue in 3% sodium tetra-borate were analyzed with a Leica DMR light microscope (Leica Microsystems). Semithin and ultrathin sections were obtained in an ultramicrotome (Leica Ultracut R, Leica Microsystem.). After staining with lead citrate and uranil acetate, ultrathin sections were examined in a JEM-1400 Plus electron microscope (JEOL GmbH, München, Germany).

## Additional Information

**How to cite this article**: Lukovic, D. *et al.* Human iPSC derived disease model of MERTK-associated retinitis pigmentosa. *Sci. Rep.* **5**, 12910; doi: 10.1038/srep12910 (2015).

## Supplementary Material

Supplementary Information
