# Supplementary material for: Human iPSC derived disease model of MERTK-associated retinitis pigmentosa
Source: Sci Rep. 2015 Aug 11;5:12910. doi: 10.1038/srep12910 (PMC4531787; doi:10.1038/srep12910)
Supplement: Supplementary Information [file srep12910-s1.pdf]

SUPPLEMENTARY INFORMATION

Manuscript title: Human iPSC derived disease model of MERTK-associated retinitis pigmentosa

Author list: Dunja Lukovic, Ana Artero Castro, Ana Belén García Delgado, María de los Ángeles Martín Bernal, Noelia Luna Peláez, Andrea Díez Lloret, Rocío Pérez Espejo, Kunka Kamenarova, Laura Fernández Sánchez, Nicolás Cuenca, Marta Cortón, Almudena Avila Fernandez, Anni Sorkio, Heli Skottman, Carmen Ayuso, Slaven Erceg & Shomi S. Bhattacharya

SUPPLEMENTARY METHODS

Karyotype analyses

To analyze genome integrity the iPSCs were passaged using accutase (1mg/mL) in Matrigel (BD, #354277) coated T25 flasks in the presence of conditioned medium. After two passages the iPSCs were sent for G-banding to the Biobank (Biobanco de Sistema Sanitario Público, Granada, Spain).

Fingerprinting

Genomic DNA from fibroblasts and iPSCs was extracted using QIAamp DNA Blood mini kit (Quiagen) in the presence of RNAse (Roche). In total, 14 fluorescently labelled microsatellite (STRs) markers including 12 STRs from the ABI PRISM Linkage Mapping Set V2.5 (Applied Biosystems Incorporated, Foster City, CA, USA) and 2 in-house designed markers were used for fingerprinting. Multiplex STR genotyping was performed using 4-6 markers per reaction using QPCR mix (ABgene, UK) and subsequently analyzed on an ABI3730xl genetic analyzer using 500 LIZ Size Standard (Applied Biosystems). Data collection and allele identification were performed using GeneScan and GeneMapper v4.0 software (Applied Biosystems).

RNA extraction and reverse transcriptase polymerase chain reaction (RT-PCR and qRT-PCR)

Cells were collected by centrifugation and total RNA was isolated with the RNeasy Mini Kit (Qiagen, Hilden, Germany) following the manufacturer´s instructions, and treated with DNase1 to remove any genomic DNA contamination. QuantiTect Reverse Transcription Kit (Qiagen) was used to carry out cDNA synthesis from 1 μg of total RNA according to the manufacturer´s instructions. The PCR reaction was performed with MyTaq Red DNA Polymerase (Bioline GmbH, Luckenwalde, Germany) with denaturation at 94ºC for 15 seconds, annealing 58ºC for 45 seconds, extension 72ºC for 45 seconds, for 30 cycles. See primer sequences in **Supplementary Table S1**. Glyceraldehydes 3-phosphate dehydrogenase (GAPDH) expression was used as a control housekeeping gene. Thereafter, PCR products were analyzed on 2% agarose gels.

For quantitative real-time PCR (qRT-PCR), the relative quantification analysis was performed using a *CFX96 RealTime* PCR Detection *system* and C1000 Thermal Cycler (Bio-Rad, Hercules, CA, USA). The PCR cycling program consisted of denaturing at 95°C for 10 minutes followed by 40 cycles of 95°C for 15 seconds and annealing/elongation at 60°C for 1 minute. The reactions were done in triplicate using TaqMan Gene Expression Master Mix and the following TaqMan probes (Applied Biosystems, Foster City, CA, USA): RPE65 (Hs01071462_m1), MITF (Hs01117294_m1), BEST1 (Hs00188249_m1), CRALBP (Hs00165632_m1), and MERTK (Hs01031973_m1) (**Supplementary Table S2**). PCR was done in triplicate and the expression of polymerase 2A (POL2A; Hs00172187_m1) was used as

endogenous control to normalize the variations in cDNA quantities from different samples. The results were analyzed using Bio-Rad CFX software and Microsoft Excel. Statistical analysis of qRT-PCR data from at least three biological replicates was calculated using Student's t-test using GraphPad Prism 5.02 (GraphPad Software).

Immunocytochemistry

Cells were washed in ice-cold PBS and fixed in 4% paraformaldehyde or 100% ice-cold methanol for 15 minutes. Fixed cells were washed twice in PBS and placed in blocking solution (3% normal goat or donkey serum and 0.5% Triton-X100 in PBS) for 1 h at room temperature. Cells were then incubated overnight at 4ºC with one of the following primary antibodies: rabbit anti-NANOG (1:400, Cell signaling), rabbit anti-OCT4 (1:400, Cell Signalling), rabbit anti-SOX2 (1:400, Cell Signaling), rabbit anti-BEST1 (1:100, Novus), rabbit anti-CRALBP (1:250, Abcam), rabbit anti-ZO1 (1:50, Invitrogen), mouse anti-MERTK (1:50, Abcam), mouse anti-Na/K-ATPase (1:100, Santa Cruz) or rabbit anti-RPE65 (1:100, Bioss). The following day, cells were washed three to five times in PBS and incubated with an appropriate secondary antibody (1:500, Invitrogen). After secondary antibody incubation, nuclei were stained with 4',6-Diamidino-2-Phenylindole, Dihydrochloride (DAPI) (Life Technologies, #D1306), washed three times in PBS. Cells were stained in plastic dishes and visualized on Olympus IX71 microscope coupled with DP72 camera. Samples grown on glass coverslips or transwell inserts were mounted using Vectashield Mounting Medium (Vector Lab, Burlingame, CA, USA) and imaged on Leica confocal microscope TCS SP5 using HCX PL APO lambda blue 63X/ 1.4 OIL objective. For live staining, anti-TRA-1-81 (StainAlive, Stemgent) was used in cultured cells without fixation according

to manufacturer's instructions. The antibodies used are listed in **Supplementary Table S3**.

Alkaline Phosphatase Staining

Alkaline phosphatase staining was carried out using Alkaline Phosphatase Staining Kit II (Stemgent, MA).

Western blot analysis

RPE cells were lysed in RIPA buffer (R0278 Sigma) containing a protease inhibitor mix (GE Healthcare), and total protein was quantified using a Bradford Reagent protein assay (B6916 Sigma-Aldrich). Protein lysates were denatured by 1X SDS Sample Buffer (70607 Novagen). The resulting samples were incubated at 97ºC for five minutes. Protein samples (30 µg) were then separated on TGX Stain-FreeTM Gels (Bio-Rad), visualized by Molecular Image Gel DocTM XR (Bio-Rad) and electroblotted onto a PVDF membrane (Trans-Blot® TurboTM Transfer Pack/ Bio-Rad). Membranes were incubated in blocking buffer (Prod#37515 Thermo Scientific) for 45 min to 1 h at room temperature, washed twice in TBS + 0.1% Tween for 5 min and incubated with primary antibody in blocking buffer overnight at 4ºC, except for β-ACTIN antibody (2 h at room temperature). Thereafter, blots were washed five times in TBS + 0.1% Tween and incubated with secondary antibody in blocking buffer for 45 min at room temperature. Blots were washed another five times and protein bands were visualized using WesternBrightTM ECL (Advansta). Primary antibodies against the following proteins were used for western blots: MERTK (rabbit monoclonal, 1:500, Abcam), BEST1 (rabbit polyclonal, 1:500, Abcam), CRALBP (mouse monoclonal, 1:1000, Abcam), and β-ACTIN (monoclonal, 1:4.000.000, Sigma-Aldrich) Anti-gamma tubulin (mouse monoclonal, 1:1000, Abcam). Mouse and rabbit secondary antibodies were obtained

from Sigma and used at a concentration of 1:20,000. The antibodies used are listed in **Supplementary Table S3**.

SUPPLEMENTARY TABLES

Supplementary Table S1.RT-PCR primer sets used for detecting the SeV genome and transgenes in cells reprogrammed using the CytoTune™ Sendai reprogramming vectors and RPE-specific genes.

Primer		Forward	Reverse	Size (bp)	Tº annealing (ºC)
Sendai virus	SeV	GGATCACTAGGTGATATCGAGC*	ACCAGACAAGAGTTTAAGGATATGTTC*	181	55
	Sox2	ATGCACCGCTACGACGTGAGCGC	AATGTATCGAAGGTGCTCAA*	451	55
	Klf	TTCCTGCATGCCAGAGGAGCCC	AATGTATCGAAGGTGCTCAA	410	55
	cMyc	TAACTGACTAGCAGGCTTGTCG*	TCCACATACAGTCCT GGATGATGATG	532	55
	Oct3/4	CCCGAAAGAGAAAGCGAACCAG	AATGTATCGAAGGTGCTCAA*	483	55
RPE	RPE-65	TGGAGTCTTTGGGGAGCCAA	CTCACCACCACACTCAGAAC	674	58
	MERTK	GGGAGATCGAGGAGTTTCTC	GATTATTACCGCCAAGGCCG	388	58
	CRALBP	GTGGACATGCTCCAGGATTC	GTTGCTGAGCAGCTCTTTGG	251	58
	BEST 1	GAATTTGCAGGTGTCCCTGT	ATCAGGAGGACGAGGAGGAT	214	58
	GAPDH	TGCACCACCAACTGCTTAGC	GGCATGGACTGTGGTCATGAG	87	53

Supplementary Table S2. TaqMan gene expression assay probe.

Gene	Assay ID
RPE65	Hs01071462_m1
MITF	Hs01117294_m1
BEST1	Hs00188249_m1
CRALBP	Hs00165632_m1
MERTK	Hs01031973_m1
POL2A	Hs00172187_m1

Supplementary Table S3. Antibodies used for western blot (W) and immunocytochemistry (ICC).

Antibody	Origin	Reference	Dilution	Size (kDa)
MERTK	Rabbit	Abcam (ab52968)	W 1:500<br>ICC 1:50	180-130
BESTROPHIN 1	Rabbit	Abcam (ab14928)<br>Novus E6-6	W 1:500<br>ICC 1:100	68
RPE65	Rabbit	Bioss (bs-9575R)	ICC 1:100	
CRALBP	Mouse	Abcam (ab15051)	W 1:1000<br>ICC 1:250	36-40
β- Actin	Mouse	Sigma (A3854)	W 1:4.000.000	40
Na/K ATPasa	Mouse	sc-21712	ICC 1:100	
ZO-1	Rabbit	INVITROGEN 617300	ICC 1:50	
F actin		Phalloidin Sigma<br>P1951	6.6µg/mL	
Oct4	Rabbit	Cell signaling C30A3	ICC 1:400	
Nanog	Rabbit	Cell signaling D73G4	ICC 1:400	
Sox2	Rabbit	Cell Signaling D6D9	ICC 1:400	
SSEA-4	Mouse	BD Pharmingen<br>560073	ICC 1:100	
TRA-1-81	Mouse	Stemgent 09-0069	1:100	

SUPPLEMENTARY FIGURES

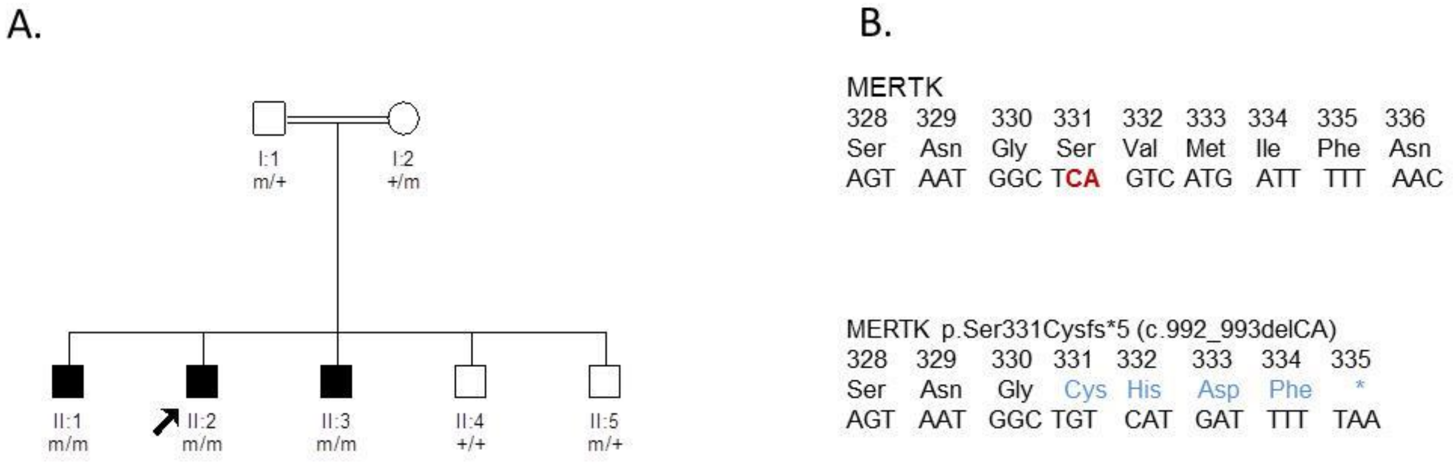

Supplementary Fig.S1.A. Pedigree of the Spanish family with mutations in the *MERTK* gene and co-segregation analysis. The MERTK genotype of each family member is represented below the individual symbol, being "+" the wild type allele and "m" mutated allele (m: c.992_993delCA; p.Ser331Cysfs*5). Nucleotide numbering reflects cDNA numbering with +1 corresponding to the A of the ATG translation initiation codon in the reference sequence NM_006343.2, according to journal guidelines (www.hgvs.org/mutnomen, accessed December 19, 2014). The initiation codon is codon 1.
B. Nucleotide sequence (NM_006343.2) and coding amino acids in MERTK surrounding Ser331. The deletion of CA at position c.992_993 results in the amino acid code frameshift and premature STOP codon (p.Ser331Cysfs*5).

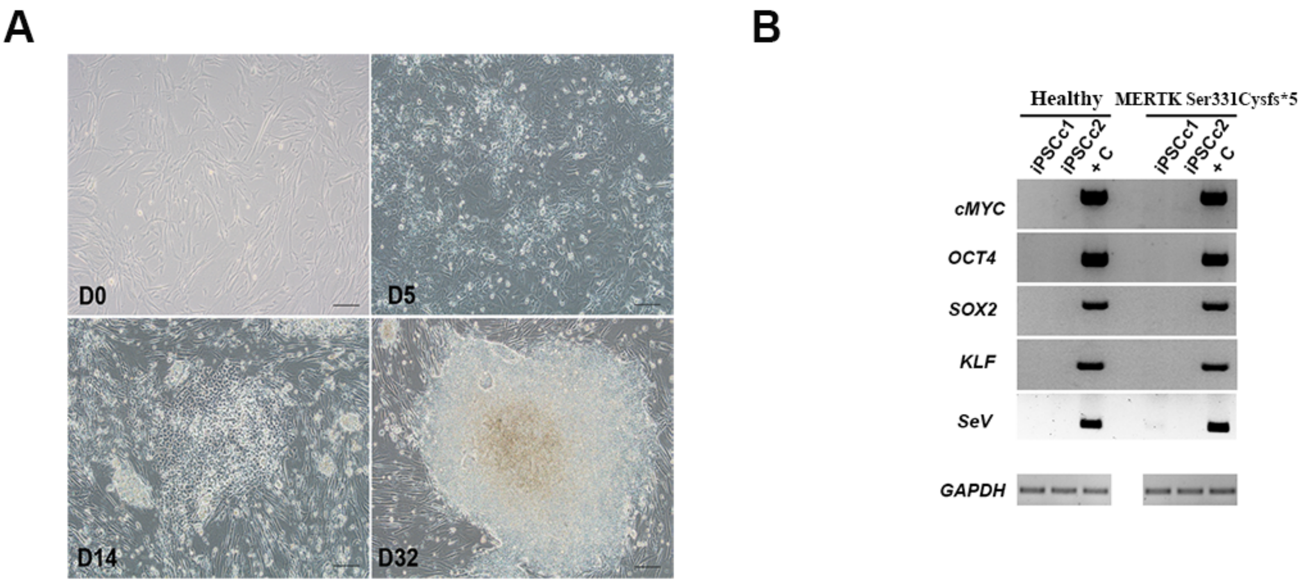

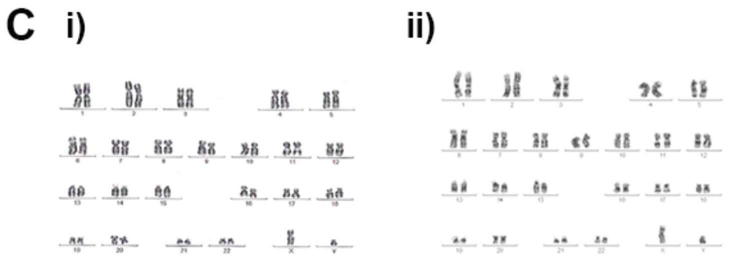

D

PANEL	MARKER	Expected size	Healthy			MERTK Ser331Cysfs*5		
			DF	iPSCc1	iPSCc2	DF	iPSCc1	iPSCc2
1	D19S572	112 - 136	119/130	119/130	119/130	130/130	130/130	130/130
	D2S2159	152-186	170/174	170/174	170/174	174/182	174/182	174/182
	D17S1533	210-226	217/217	217/217	217/217	217/217	217/217	217/217
	D14S972	201 - 211	199/203	199/203	199/203	197/197	197/197	197/197
	D8S601	223 - 236	225/225	225/225	225/225	226/234	226/234	226/234
	D9S1853	247 - 265	252/252	252/252	252/252	252/256	252/256	252/256
2	D8S1179	162 - 194	165/185	165/185	165/185	181/189	181/189	181/189
	D18S51	263	289/293	289/293	289/293	277/293	277/293	277/293
	D2S1338	165 - 205	181/193	181/193	181/193	181/181	181/181	181/181
	TH01	230 - 274	250/257	254/260	254/260	250/254	250/254	250/254
3	D16S539	148 - 172	155/159	155/159	155/159	147/164	147/164	147/164
	D19S433	199 - 221	-	-	-	199/199	199/199	199/199
	vWA	121 - 183	148/161	148/161	148/161	148/148	148/148	148/148
	D21S11	202 - 260	222/236	222/236	222/236	222/227	222/227	222/227

Supplementary Fig. S2. Reprogramming and characterization of healthy and MERTK p.Ser331Cysfs*5 fibroblasts.

A. Phase contrast images taken at day 0, 5, 14, 32 of reprogramming RP patient's fibroblasts. The cell shape changes during the reprogramming process. A typical iPSC colony (day 32) forms a defined border toward feeder cells and has refractive edges. Scale bar 200µm.
B. Expression of viral transgenes in derived iPSC lines. Two iPSC lines from each individual are analyzed at passages 7-10. The fibroblasts at day 7 after viral transduction are taken as positive control.
C. Karyotype analyses of healthy (i) and MERTK p.Ser331Cysfs*5 (ii) iPSC lines at passage 10. Both individuals have normal 46, XY karyotype.
D. Fingerprinting analyses of dermal fibroblasts and two derived iPSC lines iPSCc1 and iPSCc2 from each individual.

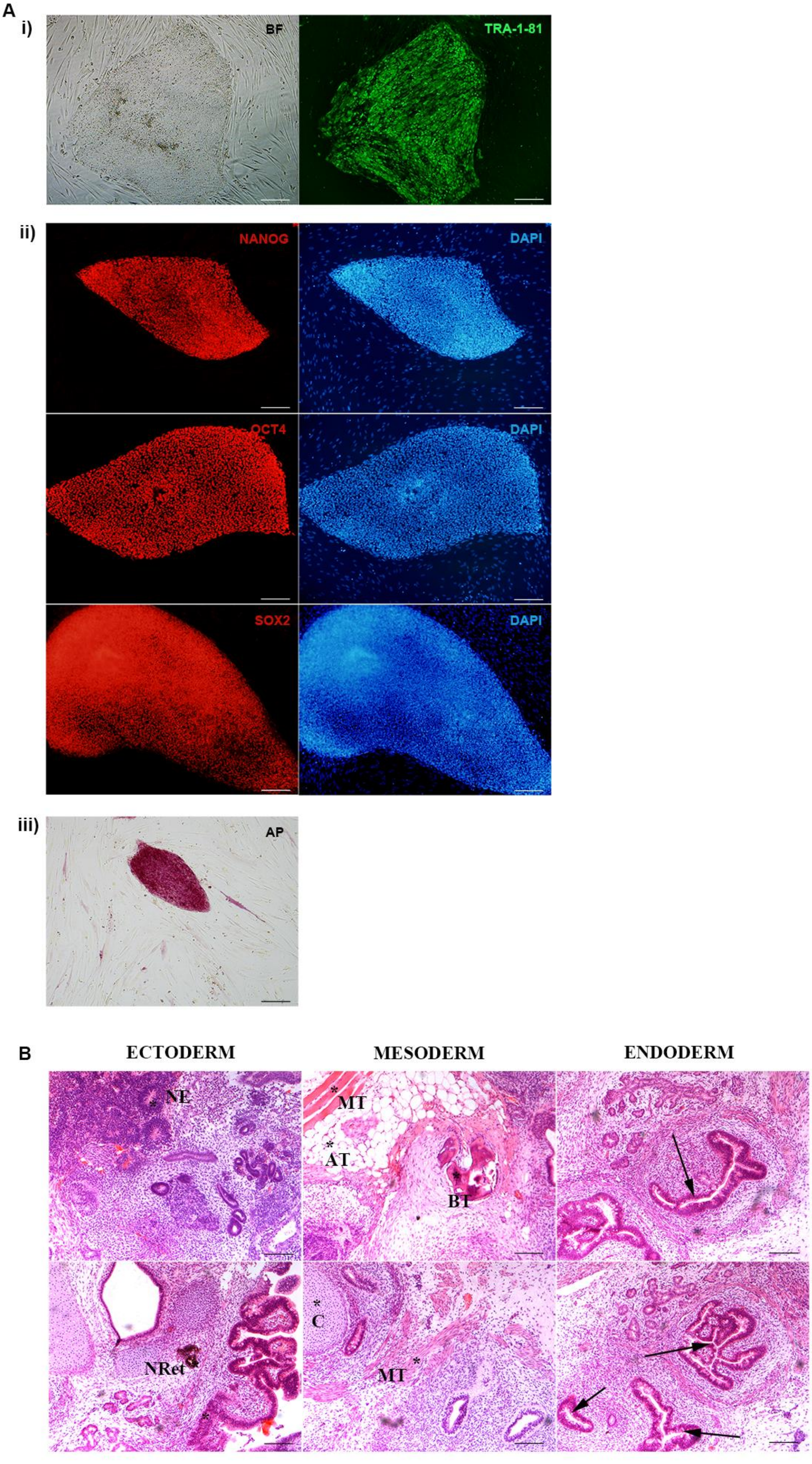
A
i)
BF
TRA-1-81
ii)
NANOG
DAPI
OCT4
DAPI
SOX2
DAPI
iii)
AP
B
ECTODERM
MESODERM
ENDODERM
NE
NRet
MT
AT
BT
C
MT

Supplementary Fig.S3. Characterization of MERTK p.Ser331Cysfs*5 iPSCs.

A. i) *In situ* staining for TRA-1-81 (bright field BF and florescence image) ii) Immunocytochemistry of pluripotency markers (NANOG, OCT4, SOX2) iii) alkaline phosphatase assay in patient's iPSC. The same markers are expressed by healthy individual iPSC (data not shown).Scale bar 200µm.

B. Teratoma induced by MERTK p.Ser331Cysfs*5 iPSCs in SCID mice. The presence of ectoderm (neural epithelium NE and retina NRet), gut endoderm (arrows) and mesoderm (bone tissue BT, adipose tissue AT, cartilage tissue CT, muscle tissue MT) in the teratoma. The same germ lineage derivatives were detected after injection of healthy individual iPSC (data not shown).Scale bar 100µm.

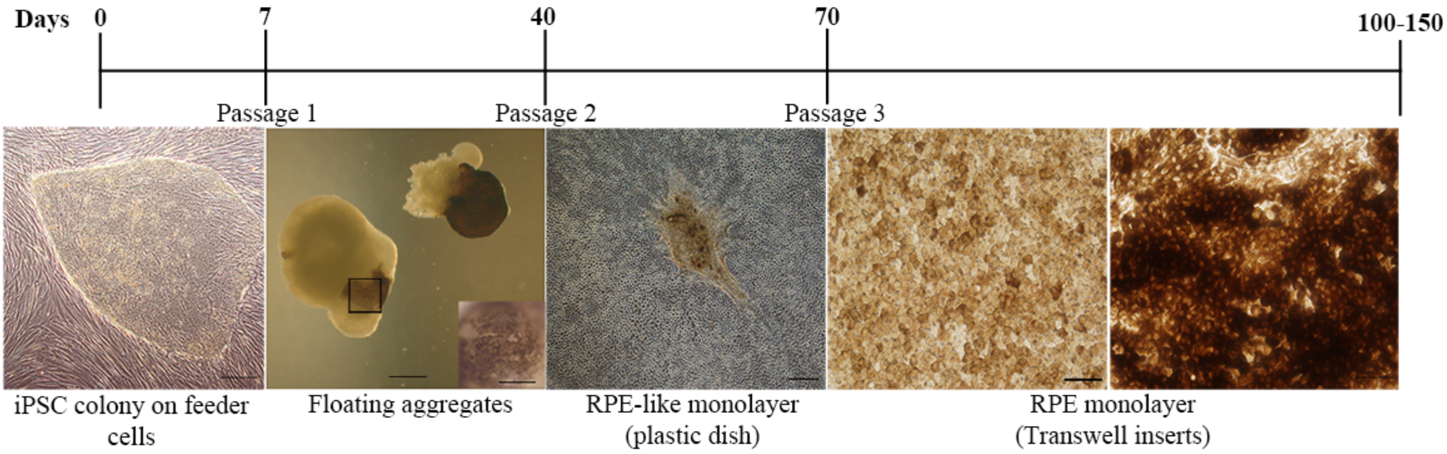

Supplemetary Fig. S4. The protocol for differentiation of iPSC into RPE cells. iPSC are grown to confluence and at around day 7 cut mechanically and cultured in low-adhesion plates until the dark patches are formed. The dark patches are microdissected, trypsinized and plated in matrigel- coated plastic plates. Once a confluent monolayer is formed, the non digested aggregates are eliminated and the cells in monolayer trypsinized, passed through a strainer and plated on transwell inserts. The cells are cultured until they reach high pigmentation. Scale bars: 200μm (iPSC colony, floating aggregates, RPE-like monolayer) and 50μm (RPE monolayer).

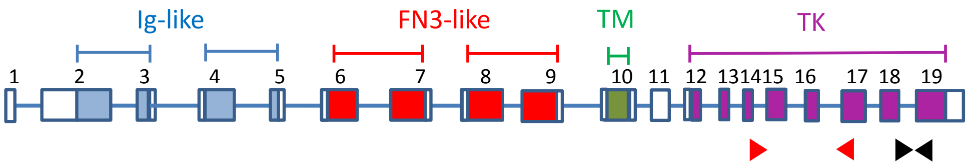

Supplementary Fig. S5. Amplicons location in relation to *MERTK* structure and MERTK domains (NP_006334.2). The 19 coding exons are depicted as rectangles and the coding regions for Ig-like, FN3-like, transmembrane (TM) and tyrosine kinase (TK) functional domains. Primers used for RT-PCR span exons 14-17 (red triangles) and qPCR probe (Hs01031973_m1) spans exons 18-19 (black triangles).

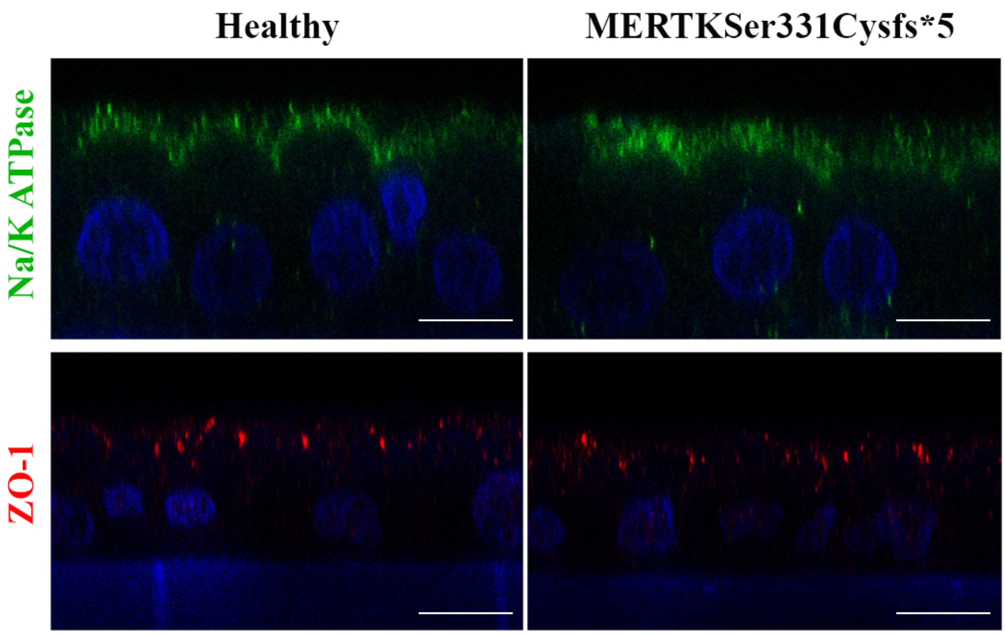

Supplementary Fig. S6. Apical and apico-lateral localization of Na^+/K^+ ATPase and ZO-1, respectively, in iPSC-RPE from healthy and MERTK p.Ser331Cysfs*5 patient. Vertical confocal sections. ZO-1 (red), Na^+/K^+ ATPase (green). Images were taken with Leica confocal microscope TCS SP5 using HCX PL APO lambda blue 63X/ 1.4 OIL objective, scale bar 10µm.

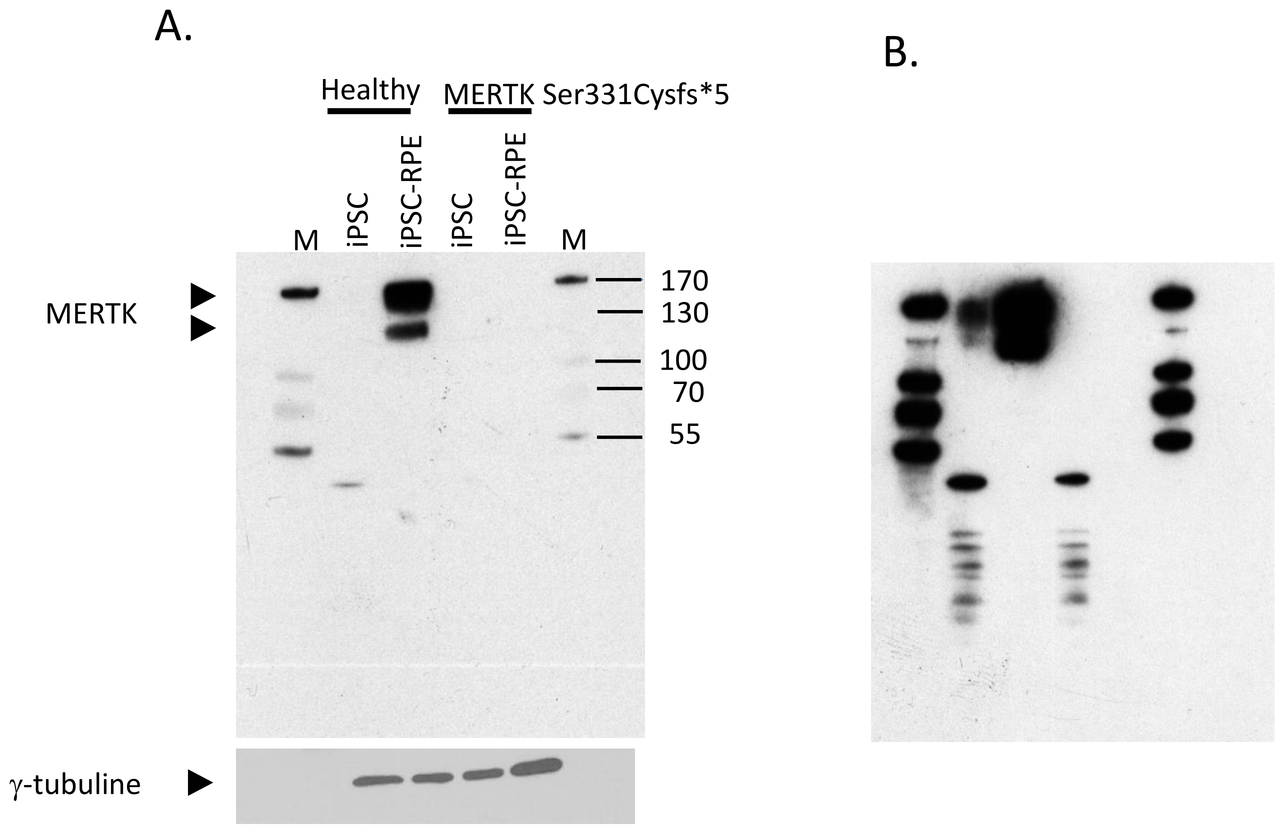

Supplementary Fig. S7. Detection of MERTK by western blot. A. iPS and iPS-RPE from healthy individual and RP patient protein extract are analyzed for the existence of truncated MERTK protein. The whole gel is demonstrated. Panel below shows γ tubulin as loading control. M: Molecular weight marker. Total protein load 80µg. Membrane exposure to the film 15 seconds. B. The same membrane exposed for 15 min.

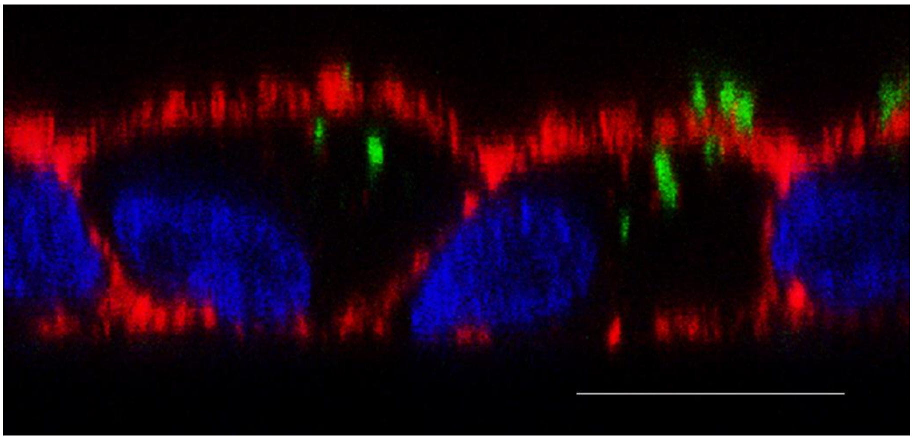

Supplementary Fig. S8. *In vitro* phagocytosis assay of photoreceptor outer segments (OS) by iPS-RPE from healthy individual: vertical confocal sections. F-actin is stained by phalloidin (red) to visualize cell morphology. OS are labeled with FITC (green) and nuclei with DAPI (blue). Images were taken with Leica confocal microscope TCS SP5 using HCX PL APO lambda blue 63X/ 1.4 OIL objective, scale bar 10µm.
